# The Utility of Troponin Dynamics in Influenza Myopericarditis: A Literature Review

**DOI:** 10.7759/cureus.60672

**Published:** 2024-05-20

**Authors:** Omar Rafa, Eric J Basile

**Affiliations:** 1 Internal Medicine, University at Buffalo, Buffalo, USA; 2 Internal Medicine, University of Florida College of Medicine, Gainesville, USA

**Keywords:** influenza virus type a, influenza b, cardiac troponin i, myo-pericarditis, cardiology

## Abstract

Influenza, typically recognized as a respiratory ailment, can manifest severe cardiac complications, notably, myocarditis and pericarditis, with potential fatal outcomes. Interestingly, influenza B demonstrates a reduced occurrence of troponin I elevation despite the risk of cardiac issues, such as isolated pericarditis. Interpreting the absence of troponin elevation as an indication of no cardiac involvement in cases of influenza B-related pericarditis may be contributing to poorer clinical outcomes. This trend may stem from the cellular tropism and unique affinity of certain influenza strains for pericardial cells rather than myocardiocytes. A thorough grasp of troponin dynamics in influenza is pivotal for customizing approaches aimed at improving clinical outcomes in myopericarditis cases.

## Introduction and background

Myocarditis and pericarditis represent inflammatory afflictions of the cardiac system, with pericarditis notably standing as the prevailing pericardial syndrome [1]. Frequently co-occurring, these maladies exhibit overlapping clinical presentations, diagnostic modalities, and therapeutic strategies [1]. Their etiological spectrum encompasses a wide array, ranging from viral and autoimmune origins to cases of idiopathic nature [2]. Clinically, the manifestation of these conditions manifests with a spectrum of severity, spanning from mild flu-like symptoms to profound cardiac decompensation and shock in both adult and pediatric myocarditis cases, with most patients typically exhibiting symptoms such as chest pain and fever [1,2]. Therapeutically, the management approach entails a multifaceted strategy, encompassing supportive measures for heart failure, utilization of inotropic agents, and cardiac medications in myocarditis cases, while pericarditis management necessitates vigilant identification of tamponade, often necessitating pericardiocentesis [2]. 

Among the myriad etiologies of myocarditis, Influenza myocarditis stands out as a rare yet potentially grave complication stemming from influenza infection. Notably, the assessment of troponin I levels in such cases presents a complex scenario, with research yielding divergent findings. Troponin I levels in these cases can vary with some studies reporting elevated levels [3,4], while others finding no elevation [5]. For example, in a case of H1N1-induced myocarditis, troponin I levels were found to be 18 ng/ml [3], while in another case of fulminant myocarditis due to H1N1, troponin I was 5 ng/ml [6]. These variations suggest that troponin I levels in influenza myocarditis cases may be influenced by factors such as the severity of the infection and the location of the involved cardiac tissue (e.g., myocardial versus pericardial involvement). Similarly, influenza pericarditis emerges as a notable phenomenon arising from influenza infections, documented in diverse case reports [7,8]. However, the depiction of pericarditis across cases exhibits significant heterogeneity. The former case does not report any troponin I levels while the latter reports a troponin I level up to 0.20 ng/mL [7,8]. Moreover, the co-occurrence of exclusive myocarditis and pericarditis in some cases further complicates the interpretation of troponin I level variations, particularly between Influenza A and B strains. Although some studies suggest more frequent troponin I elevations in Influenza A infections compared to B, the direct relationship between such elevations and myo-pericarditis remains uncertain [4]. 

The variability in troponin I levels can be further elucidated by contrasting the pathophysiologies of pericarditis and myocarditis. Pericarditis primarily affects the outer pericardial cells, whereas myocarditis reflects an inflammation of the myocardiocytes, which are closer to the endothelium layer of the heart. The anatomical location of cardiac involvement may influence troponin I elevations, with myocarditis being more probable due to its proximity to the heart's central vasculature. The literature indicates that troponin I level elevations are less frequent in influenza B, suggesting a potential higher affinity for pericardial tissue, potentially due to this layer being furthest from the endothelium, as well as the coronary vasculature. [4]. Notably, to our knowledge, formal research specifically analyzing troponin I levels in both influenza strains is lacking. Discerning the clinical utility of troponin I levels in active influenza myopericarditis cases assumes significance, as negative findings could potentially attenuate suspicions of cardiac involvement. Consequently, this paper aims to synthesize contemporary literature, juxtaposing multiple data points to elucidate the utility of troponin I levels in influenza-associated cardiac manifestations.

## Review

Elevated troponin I levels in influenza patients represent a relatively uncommon phenomenon. A comprehensive meta-analysis highlighted the infrequent nature of troponin I elevation in this cohort, occurring within a range of 0-33% of cases [9]. Moreover, the analysis highlighted a predilection for its occurrence among elderly patients burdened with comorbidities, indicating a potential susceptibility profile within this demographic. Importantly, the observed troponin I elevation tends to manifest transiently and is reversible, often correlating with the presence of cardiovascular symptoms, myocarditis, or acute ischemic episodes. Multiple etiological factors necessitate consideration within the context of influenza-related troponin I elevations. These include the perplexing variations in troponin I elevation patterns associated with each strain, challenges in distinguishing between myocarditis and pericarditis presentations in cases of influenza B, which may be potentially influenced by distinct cellular tropism mechanisms, and the concept of demand ischemia, exploring its origins and implications within the context of influenza's infectious nature. 

Influenza A versus B troponin I elevation discrepancy 

The variation in troponin I elevation patterns between influenza A and B strains bears critical significance, potentially influencing clinical decision-making regarding the necessity for further cardiac evaluation in patients with suspected cardiac involvement. In one retrospective, single-center observational study, of a total of 1,131 confirmed influenza patients, 76.2% of the infections were due to the A strain while the rest were due to the B strain [4]. Approximately 33 patients (2.9%) had an elevation of troponin I levels greater than 0.3 ng/mL and nearly 91% of them were found in type A strain instead of B, despite the latter accounting for about a quarter of cases [4]. Notably, among the subset of patients with elevated troponin I levels, a substantial proportion manifested cardiac complications, with 15 cases diagnosed with myocardial infarctions and 20 exhibiting left ventricular abnormalities [4]. This observation raises intriguing implications, suggesting a subset of patients may experience cardiac injury without evident coronary artery plaque rupture, possibly implicating myocarditis as an underlying mechanism.

Moreover, the disparity in left ventricular abnormalities outnumbering myocardial infarctions within this cohort underscores the potential involvement of myocarditis in influenza-related cardiac pathology. However, the absence of troponin I elevations in influenza B cases complicates the interpretation [7]. Indeed, a notable limitation evident in contemporary literature revolves around the correlation of troponin I levels with influenza-specific manifestations in both the pericardium and myocardium. The clinical utility of troponin I levels assumes significance in various clinical scenarios, particularly in guiding subsequent investigations for cardiac involvement. For instance, in the context of a patient presenting with influenza and concurrent elevated troponin I levels, two key factors merit consideration in directing clinical suspicion toward a cardiac cause: the magnitude of troponin elevation and the presence of chest pain symptoms. Elevated troponin I levels, especially when pronounced, invariably heighten suspicion of primary coronary involvement. However, chest pain, while indicative of potential coronary involvement, lacks specificity and may not always definitively point towards myocardial infarction.

The prior study examining troponin discrepancies between Influenza A and B strains revealed that among the 33 cases with elevated troponin I levels, only 15 exhibited actual coronary involvement, manifesting as myocardial infarction [4]. Notably, five of these cases displayed left ventricular abnormalities, indicating structural heart pathology independent of coronary involvement [4]. While not explicitly delineated in this study, such ventricular abnormalities are frequently observed in myocarditis cases, hinting at the potential for diverse cardiac manifestations beyond myocardial infarction. In this context, the utility of troponin I as a predictive marker for specific cardiac manifestations of influenza infections appears adjunctive at best. While primary coronary involvement remains a crucial consideration in cases of elevated troponin I levels in influenza cases, it is reasonable to extrapolate that isolated structural heart pathologies such as myocarditis and pericarditis may manifest independently, even in the absence of concurrent myocardial infarction. Nonetheless, it is imperative to recognize that the absence of troponin I elevation in influenza B cases does not preclude the presence of influenza cardiac-related pathology, suggesting the need for vigilance in assessing cardiac involvement across influenza subtypes. The discrepancy in troponin I elevation incidence between influenza A and B strains prompts consideration of other underlying factors such as cellular tropism. 

Cellular tropism: influenza B may be more selective for pericardium

The severity of influenza infections, including the potential for troponin I elevation, may be influenced by differences in cell tropism between influenza strains [10]. This nuanced understanding suggests a multifaceted interplay between viral strain specificity and cardiac manifestations. This study utilizes a mathematical framework to investigate the tropism of influenza A and avian-adapted influenza A strains toward specific epithelial cell surfaces [10]. Results demonstrate varying affinities for these strains, attributed to variations in sialic acid a-2,3 galactose terminated saccharides on epithelial cell surfaces. Similarly, concerning influenza B, isolated cases of pericarditis have been reported, suggesting a potential affinity for pericardial cells over myocardiocytes [7,11]. Although combined myopericarditis and exclusive myocarditis events in influenza B have been documented, the former often exhibits troponin I elevations, while the latter typically does not [8,12]. 

Indeed, one comprehensive systematic review highlights the differential cell tropism exhibited by various influenza strains within the human respiratory tract [13]. Notably, Influenza B, in contrast to influenza A and H1N1, demonstrates a predilection for infecting a diverse array of cell types, particularly those lining the upper conducting airways, which are more of the upper part of the lung [13]. This intriguing observation not only accentuates the diversity in viral tropism but also hints at potential variations in cellular affinity among different strains. However, despite the wealth of knowledge regarding respiratory cell tropism, contemporary research has yet to shed light on the specific affinity of influenza strains for pericardial versus myocardial tissues. While the hypothesis positing a correlation between isolated pericarditis cases in influenza B and the lower incidence of troponin I elevations holds clinical significance, it remains unsubstantiated by empirical evidence. Nonetheless, it's crucial to recognize the gravity of influenza B pericarditis as a potentially life-threatening complication, irrespective of troponin I levels, necessitating vigilant clinical suspicion and management protocols. The concept of cellular tropism is not unique to influenza but rather a widespread phenomenon in the realm of infectious diseases. Although much progress has been made in understanding pulmonary manifestations, the exploration of extra-pulmonary consequences, particularly myocardial and pericardial involvement, remains an area ripe for investigation and discovery. As research in this field continues to evolve, it holds the promise of uncovering novel insights into the pathogenesis of influenza-related cardiac complications, including myopericarditis. By elucidating the intricate interplay between viral tropism and tissue-specific responses, future studies may pave the way for more targeted therapeutic interventions and improved clinical outcomes in patients affected by these debilitating complications.

Regarding troponin I as a myocarditis marker, a study published in the American Heart Association Journal found elevated levels in 24 out of 26 mice with biopsy-proven myocarditis [14], aligning with expectations of direct myocardiocyte damage leading to elevated cardiac enzymes. However, in pericarditis cases, a study in the Journal of the American College of Cardiology revealed that only 32.2% of participants with pericarditis exhibited troponin I elevation [15]. This discrepancy in troponin I levels in influenza-related cardiac events may be attributed to the tropism of various strains, as explained earlier. Specifically, the affinity of influenza B strains to pericardial cells could explain isolated pericarditis events without troponin I elevation, highlighting the potential limitations of troponin I as a marker in these cases.

Keeping a wide differential with troponin I elevations in influenza patients

Nonetheless, it is crucial to comprehend the diverse origins of troponin I elevations in influenza cases, particularly those devoid of myopericarditis. Factors such as age, pre-existing cardiovascular conditions, and respiratory ailments can significantly contribute to cardiac strain, thereby resulting in troponin I elevations. Moreover, influenza-associated pneumonia has been substantiated as a catalyst for demand ischemia, as indicated by numerous case studies [16]. In severe scenarios, influenza-related pneumonia can progress to acute respiratory failure, hemolytic anemia, acute renal failure, and myocarditis [17]. Noteworthy is pneumonia's prominence as the primary influenza complication, frequently culminating in pulmonary inflammation [18]. Transient pulmonary inflammation alone can precipitate troponin I elevations. Hence, it is imperative to keep demand ischemia in mind when assessing influenza-positive patients with elevated troponin I levels.

An essential consideration lies in the strong association between troponin I elevations and influenza infections, encompassing not only demand ischemia but also primary coronary artery plaque rupture or myocardial infarction. Indeed, influenza infection consistently emerges as a significant risk factor for acute myocardial infarctions, attributed to its capacity to incite inflammation, provoke plaque rupture, and instigate prothrombotic events, as corroborated by multiple studies [19,20]. This association notably intensifies during severe infections and can exacerbate pre-existing cardiovascular conditions [20]. Moreover, influenza vaccination emerges as a potent tool in mitigating the incidence, morbidity, and mortality of myocardial infarctions among patients with coronary artery disease [21]. However, despite the robust evidence supporting the efficacy of influenza vaccination in high-risk populations, vaccination rates remain suboptimal [19,20]. Thus, advocating for widespread influenza vaccination as a preventive measure for cardiovascular disease, including myocardial infarctions, remains imperative.

The connection between Influenza B and myocardial infarctions presents a complex scenario, lacking definitive elucidation within contemporary literature. Specifically, the precise strain responsible for this correlation remains undifferentiated in existing research. Nevertheless, a plausible inference can be drawn, positing that predominant myocardial involvement may exhibit a heightened propensity for myocardial infarctions compared to pericardial involvement, given its closer proximity to the coronary arteries. Moreover, given the acknowledged phenomenon of cellular tropism between various influenza strains and the noted dissimilarity in troponin I elevations between influenza A and B, there arises a pressing need for further investigation into the role of troponin I in predicting cardiac involvement, encompassing not only structural myocardial implications as previously explored but also coronary involvement, particularly among patients afflicted with influenza B [4,10]. This call for deeper inquiry reflects the intricate and multifaceted nature of the interaction between influenza infection, myocardial infarctions, and troponin I elevations, underlining the complexity inherent in cardiovascular complications within the realm of influenza. As such, navigating this intricate landscape necessitates a sophisticated and nuanced approach to patient management and risk assessment, one that recognizes the dynamic interplay between viral infection, cardiac pathology, and biomarker dynamics. 

Expanding upon the established association between myocardial infarction and influenza incidence, a crucial aspect of patient management lies in navigating symptoms to guide appropriate interventions. Considering the pivotal role of troponin I utility in influenza infections for discerning cardiac-origin culprit diagnosis, it becomes imperative to compare the symptomatology of pulmonary manifestations stemming from influenza with that of myocardial infarctions. Notably, research has elucidated that both acute pulmonary diseases and myocardial infarction can manifest with overlapping symptoms, including dyspnea, chest pain, and fever [22-24]. This principle extends beyond influenza to encompass a broader spectrum of pulmonary pathologies that can mimic the presentation of myocardial infarctions. In addition to influenza, various pulmonary conditions may manifest with symptoms resembling those of myocardial infarctions. For instance, conditions such as pneumonia, pulmonary embolism, and acute respiratory distress syndrome (ARDS) can present with symptoms such as chest pain, dyspnea, and fever, which overlap with those commonly associated with myocardial infarctions [24]. This convergence of symptoms poses a significant challenge, potentially leading to misdiagnosis, as exemplified by several cases of pulmonary embolism masquerading as a myocardial infarction [22,24].

This scenario introduces a theoretical quandary wherein the resemblance between symptoms of pulmonary conditions and those of myocardial infarctions may obscure clinical judgment, posing challenges in accurately diagnosing and managing patients. However, the complexity deepens when considering that primary myocardial infarctions rarely account for elevated troponin I levels, as indicated by a large retrospective chart review [25]. Among the 23,731 emergency room visits analyzed, only a minority were classified as type I myocardial infarctions (11%), with a substantial proportion attributed to other cardiovascular diagnoses (34%) or noncardiovascular conditions (55%) [25]. Hence, the rarity of primary myocardial infarctions amidst presentations of generic chest pain and dyspnea underscores the importance of careful differential diagnosis, particularly given the potential overlap with postmyocardial infarction syndrome, which can manifest as pneumonia with bloody expectoration, as observed [26]. In the realm of symptomology and troponin I levels in patients with influenza, a nebulous interplay emerges between pulmonary and primary myocardial infarction incidences. Amidst this uncertainty, one consistent observation is the heightened incidence of demand ischemia, predominantly defined as non-coronary plaque disruption, as a cause of troponin I elevations in patients with influenza. This disparity underscores the need for meticulous differential diagnosis and highlights the plausible pathogenetic link between respiratory symptoms and the onset of myocardial infarction. Consequently, a structured approach toward evaluating chest pain in individuals with influenza necessitates a thorough assessment to rule out underlying myocardial infarctions, thereby ensuring appropriate clinical management and optimizing patient outcomes.

A deeper dive into influenza B mortality: cardiac and non-cardiac 

In terms of our literature review, there remains an unexplored realm within contemporary literature concerning mortality rates among patients with influenza who exhibit elevated troponin levels. Despite this gap, independent meta-analyses have emerged, shedding illuminative insights into the topic, particularly focusing on patients admitted with elevated troponin I levels devoid of a primary cardiac diagnosis. In one such meta-analysis, consisting of 27 studies, notable findings surface, showcasing a significant association between elevated troponin levels and heightened mortality rates [27]. Specifically, the meta-analysis elucidates a compelling correlation between elevated troponin levels and increased in-hospital mortality, as well as a heightened risk of mortality within the first 30 days post-admission [27]. Furthermore, the analysis unearths a noteworthy escalation in the risk of long-term mortality, spanning a six-month period, among patients with elevated troponin levels. 

While this evidence solidifies the independent correlation between elevated troponin levels and heightened mortality risks in non-cardiac patients, a specific investigation into patients with influenza and elevated troponin I levels has yet to be undertaken. Despite the absence of dedicated studies, numerous case reports have documented severe and fatal outcomes associated with this cohort [7,8]. The underlying causes of mortality in these cases are diverse, potentially stemming from various factors, including demand ischemia, respiratory failure, exacerbation of primary coronary plaque ruptures, and structural heart involvement such as myo-pericarditis [16-18]. Moreover, the emergence of isolated pericarditis cases linked to influenza B, without concurrent troponin I elevations, introduces another layer of complexity to mortality rates in influenza-associated cardiac pathologies. While elevated troponin I levels have been identified as an independent predictor of mortality in hospitalized patients without primary cardiac involvement, the presence of influenza B may serve as an additional independent predictor of mortality irrespective of troponin I levels.

This assertion is further underscored by a large observational study spanning from 1979 to 2001, which analyzed direct deaths attributed to influenza on an annual basis, revealing a staggering figure of 41,000 deaths per year [28]. Notably, however, the study did not specifically identify influenza B as an independent predictor of mortality nor did it elaborate on any specific myopericarditis cases related to those deaths. This discrepancy further supports the potential limitations of troponin I levels as prognostic indicators in patients with influenza B, as the virus has been shown to precipitate cardiac events, including pericarditis, without concurrent troponin I elevations. Moreover, influenza B is implicated in the onset of various life-threatening conditions unrelated to cardiac pathology, further diminishing the prognostic value of troponin I levels in this subset of patients. Thus, while troponin I levels remain a valuable marker in certain clinical contexts, their utility in predicting outcomes in patients with influenza B warrants careful consideration amidst the broader spectrum of potential morbidity and mortality factors associated with the virus.

## Conclusions

When examining troponin I levels in patients with influenza, clinicians must acknowledge the potential limitations in its utility for providing precise guidance for diagnosing cardiac-related issues. The variability in troponin I levels stems from several factors, encompassing pulmonary manifestations, coronary plaque involvement, and potentially influenza B's cellular tropism in terms of potential predilection for pericardial rather than myocardial cells leading to isolated pericarditis without concurrent elevations in troponin I levels. Each of these factors contributes to the complex landscape of cardiac manifestations associated with influenza infection.
